# The Multi-Cover Persistence of Euclidean Balls

**DOI:** 10.1007/s00454-021-00281-9

**Published:** 2021-03-31

**Authors:** Herbert Edelsbrunner, Georg Osang

**Affiliations:** Institute of Science and Technology Austria, Am Campus 1, 3400 Klosterneuburg, Austria

**Keywords:** Delaunay mosaics, Hyperplane arrangements, Discrete Morse theory, Zigzag modules, Persistent homology

## Abstract

Given a locally finite $X \subseteq {{{\mathbb {R}}}}^d$ and a radius $r \ge 0$, the *k*-*fold cover* of *X* and *r* consists of all points in ${{{\mathbb {R}}}}^d$ that have *k* or more points of *X* within distance *r*. We consider two filtrations—one in *scale* obtained by fixing *k* and increasing *r*, and the other in *depth* obtained by fixing *r* and decreasing *k*—and we compute the persistence diagrams of both. While standard methods suffice for the filtration in scale, we need novel geometric and topological concepts for the filtration in depth. In particular, we introduce a rhomboid tiling in ${{{\mathbb {R}}}}^{d+1}$ whose horizontal integer slices are the order-*k* Delaunay mosaics of *X*, and construct a zigzag module of Delaunay mosaics that is isomorphic to the persistence module of the multi-covers.

## Introduction

The work in this paper is motivated by density fluctuations in point configurations. These fluctuations can be large—and the task may be the identification of regions with a prescribed density profile—or they can be small—and the goal may be to pick up subtle variations. For example, we may want to quantify local defects in lattice configurations or describe long-range differences between similar configurations, such as the *face-centered cubic* (FCC) lattice and the *hexagonal close-packed* (HCP) configuration in ${{{\mathbb {R}}}}^3$. While both give densest sphere packings in ${{{\mathbb {R}}}}^3$, physical particle systems prefer to settle in the FCC configuration. The reason for this preference is not well understood. Our quantification of the long-range effects of density differences discriminates between the two configurations and this way sheds light on this phenomenon.

Using standard methods from computational geometry and topology, we describe mathematical and computational tools to quantify density fluctuations. Our work is closely related to the *distance to a measure* introduced in [6]. As demonstrated in a follow-up paper [14], this distance can be approximated using the *order*-*k* *Voronoi tessellation* of the configuration, a concept introduced in the early days of computational geometry [20], but see also [11, 16]. Order-*k* Voronoi tessellations are also at the core of our work:

1. Given a locally finite set $X \subseteq {{{\mathbb {R}}}}^d$, we introduce a rhomboid tiling in ${{{\mathbb {R}}}}^{d+1}$ whose horizontal slices at integer depths are the geometric duals of the order-*k* Voronoi tessellations.

We call these duals the *order-k Delaunay mosaics* of *X*. The tiling clarifies the structure of individual mosaics and the relationship between them. Restricting the order-*k* Voronoi tessellation to the *k*-*fold cover* of the balls with radius $r\ge 0$ centered at the points in *X*, we get a subcomplex of the order-*k* Delaunay mosaic; see [15] for the introduction of this concept for statistical purposes in two dimensions. Our second result makes use of the family of such subcomplexes obtained by varying the scale:

2. Fixing *k* and varying *r*, we compute the persistence diagram of the density fluctuations from the filtration of order-*k* Delaunay mosaics of *X*.

The ingredients for our second result are standard, but to get the actual results, we needed an implementation of the order-*k* Delaunay mosaic algorithm, which we developed based on the rhomboid tiling. In contrast to [2, 19], this gives a simple implementation, which we will describe elsewhere. Using this software in ${{{\mathbb {R}}}}^3$, we find that the FCC and HCP configurations have the same persistence diagram for $k = 1,2,3$ but different persistence diagrams for $k = 4,5$. Our third result is an algorithm for the persistence of the multi-covers obtained by varying the depth:

3. Fixing *r* and varying *k*, we compute the persistence diagram of the filtration of multi-covers from the rhomboid tiling of *X*.

Several innovative adaptations of the standard approach to persistence are needed to get our third result. The main challenge is the combinatorial difference of the Delaunay mosaics from one value of *k* to the next. Here we use the rhomboid tiling to establish a zigzag module whose persistence diagram is the same as that of the filtration of multi-covers. We get the persistence diagram using the algorithm in [4, 5]. Our work is also related to the study of multi-covers based on Čech complexes in [21]. While the relation between the different Čech complexes is simpler than that between the Delaunay mosaics, their explosive growth for increasing radius leads to algorithms with prohibitively long running time.

*Outline*   Section 2 describes the rhomboid tiling in ${{{\mathbb {R}}}}^{d+1}$ that encodes the Delaunay mosaics of all orders of a locally finite set in ${{{\mathbb {R}}}}^d$. Section 3 relates the *k*-fold covers with the order-*k* Delaunay mosaics and introduces radius functions on the rhomboid tiling and the mosaics. Section 4 introduces slices of a tiling at half-integer depths and explains how they are used to compute the persistence diagram in depth. Section 5 concludes the paper.

## Rhomboid Tiling

Given a locally finite set in ${{{\mathbb {R}}}}^d$, we are interested in the collection of Delaunay mosaics of all orders. Assuming the set is in general position, there exists a rhomboid tiling in ${{{\mathbb {R}}}}^{d+1}$ such that the Delaunay mosaics are horizontal slices of the tiling. This section introduces the tiling and proves the relation to the Delaunay mosaics.

*Rhomboid tiling*   Let $X \subseteq {{{\mathbb {R}}}}^d$ be locally finite and in general position. Every $(d-1)$-dimensional sphere, *S*, in ${{{\mathbb {R}}}}^d$ partitions *X* into the points *inside*, *on*, and *outside* *S*. We call this the *ordered three-partition* of *X* defined by *S*, and denote it as $X = {{\,\mathrm{In}\,}}{S} \cup {{\,\mathrm{On}\,}}{S} \cup {{\,\mathrm{Out}\,}}{S}$. By the assumption of general position, we have ${|{{{{\,\mathrm{On}\,}}{S}}}|}\le d+1$, but there are no a priori upper bounds on the sizes of the other two sets.

We map each ordered three-partition defined by a $(d-1)$-sphere, *S*, to a parallelepiped in ${{{\mathbb {R}}}}^{d+1}$, which we call the *rhomboid* of *S*, denoted ${{\,\mathrm{rho}\,}}{S}$. To define it, we write $y_x = (x, -1) \in {{{\mathbb {R}}}}^{d+1}$, for every $x \in X$, and $y_Q = \sum _{x \in Q} y_x$ for every $Q \subseteq X$. The $(d+1)$-st coordinate of $y_Q$ is therefore $- {|{Q}|}$, and we call ${|{Q}|}$ the *depth* of the point. With this notation, ${{\,\mathrm{rho}\,}}{S} = \mathrm{conv\,}{ \{ y_Q \mid {{\,\mathrm{In}\,}}{S} \subseteq Q \subseteq {{\,\mathrm{In}\,}}{S} \cup {{\,\mathrm{On}\,}}{S} \}}$. Equivalently, ${{\,\mathrm{rho}\,}}{S}$ is the rhomboid spanned by the vectors $y_x$, with $x \in {{\,\mathrm{On}\,}}{S}$, and translated along $y_{{{\,\mathrm{In}\,}}{S}}$. Its dimension is the number of spanning vectors, ${|{{{{\,\mathrm{On}\,}}{S}}}|}$. Observe that every face of ${{\,\mathrm{rho}\,}}{S}$ is again the rhomboid defined by a sphere. To see this, we note that for every ordered partition of the points on *S* into three sets, ${{\,\mathrm{On}\,}}{S} = {{O}_{in }}\cup {{O}_{on }}\cup {{O}_{out }}$, there is a sphere $S'$ with ${{\,\mathrm{In}\,}}{S'} = {{\,\mathrm{In}\,}}{S} \cup {{O}_{in }}$, ${{\,\mathrm{On}\,}}{S'} = {{O}_{on }}$, and ${{\,\mathrm{Out}\,}}{S'} = {{\,\mathrm{Out}\,}}{S} \cup {{O}_{out }}$. There are $3^{|{{{{\,\mathrm{On}\,}}{S}}}|}$ such ordered partitions, and each corresponds to a face of ${{\,\mathrm{rho}\,}}{S}$.

**Fig. 1:**
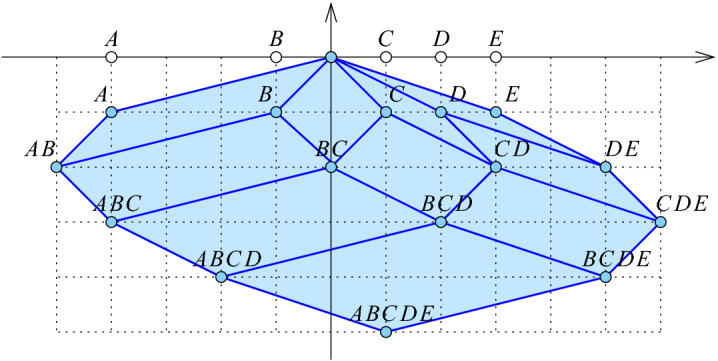
The rhomboid tiling of five points on the real line. For example, the upper left 2-dimensional rhomboid defined by $( \emptyset , \{A,B\}, \{C,D,E\} )$ is the convex hull of the points $y_\emptyset$, $y_A$, $y_B$, and $y_{\{A,B\}}$. The horizontal line at depth *k* intersects the tiling in a geometric realization of the order-*k* Delaunay mosaic of the five points

By definition, the *rhomboid tiling* of *X*, denoted ${{\,\mathrm{Rho}\,}}{X}$, is the collection of all rhomboids defined by spheres; see Fig. 1. As suggested by the figure, the ordered three-partition $(\emptyset , \emptyset , X)$ is mapped to the origin of ${{{\mathbb {R}}}}^{d+1}$. We claim the following properties.

### Theorem 2.1

(rhomboid tiling)  Let $X \subseteq {{{\mathbb {R}}}}^d$ be locally finite and in general position. Then

(i): ${{\,\mathrm{Rho}\,}}{X}$ is dual to an arrangement of hyperplanes in ${{{\mathbb {R}}}}^{d+1}$;
(ii): ${{\,\mathrm{Rho}\,}}{X}$ is the projection of the boundary of a zonotope in ${{{\mathbb {R}}}}^{d+2}$;
(iii): the horizontal slice of ${{\,\mathrm{Rho}\,}}{X}$ at depth *k* is the order-*k* Delaunay mosaic of *X*.

Note that claim (ii) in Theorem 2.1 implies that the rhomboid tiling is a geometric realization of the dual of the arrangement in ${{{\mathbb {R}}}}^{d+1}$, that is: its rhomboids intersect in common faces but not otherwise. The remainder of this section proves the three claims. To keep the proofs self-contained, we will define hyperplane arrangements and order-*k* Delaunay mosaics before we use them. We refer to [7] for additional information on their relation to point configurations.

**Fig. 2:**
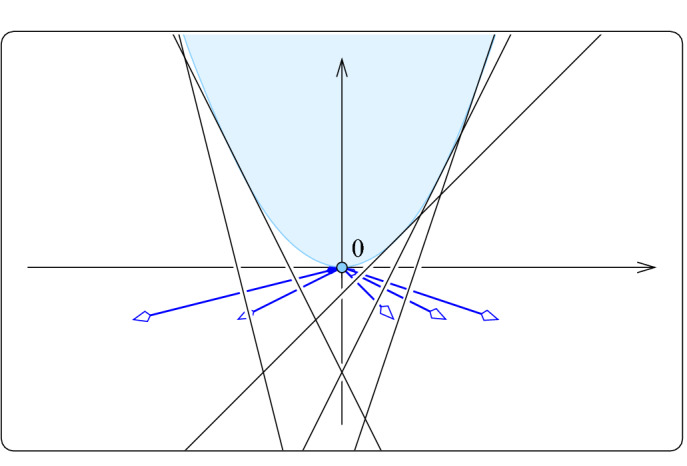
A portion of the arrangement formed by the lines (hyperplanes) that are the graphs of the $f_x$, with $x \in X$. These lines are tangent to the paraboloid and normal to the vectors $y_x = (x, -1)$. The topmost chamber contains the paraboloid

Proof of (i): hyperplane arrangement For each point $x \in X$, write $f_x :{{{\mathbb {R}}}}^d \rightarrow {{{\mathbb {R}}}}$ for the affine map defined by $f_x (p) = {\langle p , x \rangle } - {\Vert {x}\Vert }^2/2 = ( {\Vert {p}\Vert }^2 - {\Vert {p}-{x}\Vert }^2 ) / 2$. The graph of $f_x$ is a hyperplane in ${{{\mathbb {R}}}}^{d+1}$ that is tangent to the paraboloid consisting of the points $(p,z) \in {{{\mathbb {R}}}}^d \times {{{\mathbb {R}}}}$ that satisfy $z = {\Vert {p}\Vert }^2/2$. The collection of such hyperplanes decomposes ${{{\mathbb {R}}}}^{d+1}$ into convex cells, which we call the *hyperplane arrangement* of *X*, denoted ${{\,\mathrm{Arr}\,}}{X}$; see Fig. 2. The *cells* in the arrangement are intersections of hyperplanes and closed half-spaces. More formally, for each cell there is an ordered three-partition $X = {{X}_{in }}\cup {{X}_{on }}\cup {{X}_{out }}$ such that the cell consists of all points $(p, z) \in {{{\mathbb {R}}}}^d \times {{{\mathbb {R}}}}$ that satisfy

1 $$\begin{aligned} z&\le f_x (p)\qquad \text {if}\quad x \in {{X}_{in }}, \end{aligned}$$

2 $$\begin{aligned} z&= f_x (p)\qquad \text {if}\quad x \in {{X}_{on }}, \end{aligned}$$

3 $$\begin{aligned} z&\ge f_x (p) \qquad \text {if}\quad x \in {{X}_{out }}. \end{aligned}$$

Since *X* is assumed to be in general position, the dimension of the cell is $i = d+1-{|{{{X}_{on }}}|}$. Turning the non-strict into strict inequalities, we get the interiors of the cells, which partition ${{{\mathbb {R}}}}^{d+1}$. We refer to the *i*-dimensional cells as *i*-*cells* and to the $(d+1)$-cells as *chambers*.

Importantly, there is a bijection between the cells of ${{\,\mathrm{Arr}\,}}{X}$ and the rhomboids in ${{\,\mathrm{Rho}\,}}{X}$. To see this, map a point (*p*, *z*) in the interior of a cell to the sphere *S* with center *p* and squared radius $r^2 = \max { \{0, {\Vert {p}\Vert }^2 - 2z \}}$. Using the definition of $f_x$, we observe that ${{\,\mathrm{In}\,}}{S} = {{X}_{in }}$, ${{\,\mathrm{On}\,}}{S} = {{X}_{on }}$, and ${{\,\mathrm{Out}\,}}{S} = {{X}_{out }}$. We can reverse the map, and while this will not reach the points with ${\Vert {p}\Vert }^2 - 2z < 0$, these points all belong to the chamber of the ordered three-partition $(\emptyset , \emptyset , X)$. This establishes the bijection between the cells and the rhomboids. This bijection reverses dimensions and preserves incidences, which justifies that we call it a *duality* between the rhomboid tiling and the hyperplane arrangement. This completes the proof of (i) in Theorem 2.1. $\square$

Proof of (ii) We recall that a *zonotope* is a special convex polyhedron, namely one obtained by taking the Minkowski sum of finitely many line segments. The zonotope of interest is constructed from the line segments that connect the origin to the points $v_x = (x, -1, {\Vert {x}\Vert }^2/2) \in {{{\mathbb {R}}}}^{d+2}$, with $x \in X$. Note that these line segments project to the vectors $y_x = (x, -1)$ used to build the rhomboid tiling. By construction, $y_x$ is normal to the graph of $f_x$, which is the zero set of $F_x :{{{\mathbb {R}}}}^{d+1} \rightarrow {{{\mathbb {R}}}}$ defined by $F_x(q) = {\langle q , y_x \rangle } - {\Vert {x}\Vert }^2/2$; see Fig. 2. Adding a $(d+2)$-nd coordinate, *w*, we introduce $G_x :{{{\mathbb {R}}}}^{d+2} \rightarrow {{{\mathbb {R}}}}$ defined by $G_x (q,w) ={\langle q , y_x \rangle } + w{\Vert {x}\Vert }^2/2$. Its zero-set is normal to $v_x$, the restriction of $G_x^{-1}(0)$ to $w=-1$ is the zero-set of $F_x$, and $G_x (0) = 0$. In other words, if we identify ${{{\mathbb {R}}}}^{d+1}$ with the hyperplane $w = -1$ in ${{{\mathbb {R}}}}^{d+2}$, then the zero-sets of the $G_x$ intersect ${{{\mathbb {R}}}}^{d+1}$ in ${{\,\mathrm{Arr}\,}}{X}$ and they all pass through the origin in ${{{\mathbb {R}}}}^{d+2}$.

By construction, the thus defined zonotope is dual to the arrangement of hyperplanes $G_x^{-1}(0)$ for $x \in X$. Therefore, the antipodal face pairs of the zonotope correspond dually to the cells of ${{\,\mathrm{Arr}\,}}{X}$, provided we interpret the arrangement projectively, which means we combine antipodal pairs of unbounded cells; see also [7, Sect. 1.7]. We get a more direct dual correspondence by projecting the lower part of the boundary of the zonotope to ${{{\mathbb {R}}}}^{d+1}$. By choice of the line segments, the vertices on this side project vertically to the vertices of ${{\,\mathrm{Rho}\,}}{X}$, and since both are dual to ${{\,\mathrm{Arr}\,}}{X}$, we conclude that ${{\,\mathrm{Rho}\,}}{X}$ is the projection of this side of the zonotope. This completes the proof of (ii). $\square$

Proof of (iii): Delaunay mosaics We begin with some definitions. The *Voronoi domain* of $Q \subseteq X$ is ${{\,\mathrm{dom}\,}}{Q} = \{p \in {{{\mathbb {R}}}}^d \mid {\Vert {p}-{x}\Vert } \le {\Vert {p}-{y}\Vert }\ \hbox { for all}\ x \in Q,\,y \in X \setminus Q \}$. Its *order* is ${|{Q}|}$. For each Voronoi domain, there is a chamber in ${{\,\mathrm{Arr}\,}}{X}$ that projects vertically to the domain. Indeed, the chamber is defined by the ordered three-partition $X = {{X}_{in }}\cup {{X}_{on }}\cup {{X}_{out }}$ with ${{X}_{in }}= Q$, ${{X}_{on }}= \emptyset$, and ${{X}_{out }}= X \setminus Q$. For each positive integer *k*, the *order-k Voronoi tessellation* is ${{\,\mathrm{Vor}\,}}_{k}{X}=\{{{\,\mathrm{dom}\,}}{Q} \mid {|{Q}|} = k \}$. We can construct it by projecting all chambers whose ordered three-partitions satisfy ${|{{{X}_{in }}}|}=k$ and ${|{{{X}_{on }}}|} = 0$; see [7, Chap. 13] or [10]. These chambers correspond to the vertices of the rhomboid tiling at depth *k*. Since ${{\,\mathrm{Rho}\,}}{X}$ is dual to ${{\,\mathrm{Arr}\,}}{X}$, we get the dual of the Voronoi tessellation by taking the slice $z = -k$ of ${{\,\mathrm{Rho}\,}}{X}$. However, the dual of the order-*k* Voronoi tessellation is precisely the order-*k* Delaunay mosaic [1]. This completes the proof of (iii). $\square$

**Fig. 3:**
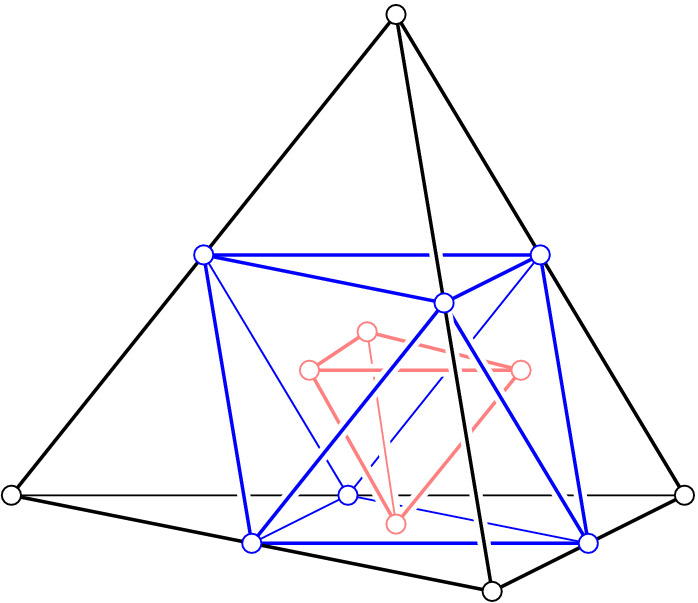
The convex hulls of the barycenters of the $(j-1)$-faces of the tetrahedron. From *outside in*: the tetrahedron for $j=1$, the octahedron for $j=2$, and another tetrahedron for $j=3$

We see that the cells of ${{\,\mathrm{Del}\,}}_{k}{X}$ are special slices of the rhomboids. Combinatorially, they are equivalent to slices of the unit cube that are orthogonal to the main diagonal and pass through non-empty subsets of the vertices. For the $(d+1)$-cube, there are $d+2$ such slices, which we index from 0 to $d+1$. The *j*-th slice passes through $$\left( {\begin{array}{c}d+1\\ j\end{array}}\right) $$ vertices, so we have a vertex for $j = 0,d+1$ and a *d*-simplex for $j = 1,d$. To describe these slices in general, let $U_{d+1}$ be the $d+1$ unit coordinate vectors. The *j*-th slice is the convex hull of the points $\sum _{u\in Q} u$ with $$Q \in \left( {\begin{array}{c}U_{d+1}\\ j\end{array}}\right) $$, in which the empty sum is $(0, 0) \in {{{\mathbb {R}}}}^d \times {{{\mathbb {R}}}}$, by convention. To get an intuition, it might be easier to divide the sums by *j*, in which case the *j*-th slice is the convex hull of the barycenters of the $(j-1)$-faces of the standard *d*-simplex; see Fig. 3.

## Multi-Covers

In this section, we exploit the rhomboid tiling to shed light on the filtration of multi-covers we get by varying the radius. The main new insight is that the discrete function on the Delaunay mosaic that encodes this filtration is a relaxation of a standard discrete Morse function. We begin with a formal introduction of the multi-covers.

*k*-*fold cover*   Let $X \subseteq {{{\mathbb {R}}}}^d$ be locally finite. Given a radius $r \ge 0$, the *k*-*fold cover* of *X* and *r* consists of all points $p \in {{{\mathbb {R}}}}^d$ for which there are *k* or more points $x \in X$ with ${\Vert {x}-{p}\Vert } \le r$, or in other words, the points $p\in {{{\mathbb {R}}}}^d$ that are covered by at least *k* of the balls of radius *r* around the points $x \in X$. Denoting this set by ${\mathrm{Cover}_{k}{({X},{r})}}$, we have

4 $$\begin{aligned} {\mathrm{Cover}_{k}{({X},{r})}}&\subseteq {\mathrm{Cover}_{k}{({X},{s})}} ,\end{aligned}$$

5 $$\begin{aligned} {\mathrm{Cover}_{k}{({X},{r})}}&\subseteq {\mathrm{Cover}_{\ell }{({X},{r})}} , \end{aligned}$$

whenever $r \le s$ and $\ell \le k$. We are interested in computing the persistent homology of the multi-covers, both in the direction of increasing radius and in the direction of decreasing order. To do so, we represent the covers by complexes, namely by subcomplexes of the Delaunay mosaics. Varying the radius, we get a nested sequence of subcomplexes of the order-*k* Delaunay mosaic, and the persistent homology can be computed with standard methods; see e.g. [8, Chap. VII]. Varying the order, on the other hand, we get subcomplexes of different Delaunay mosaics, and we need a novel algorithm to compute persistent homology.

**Fig. 4:**
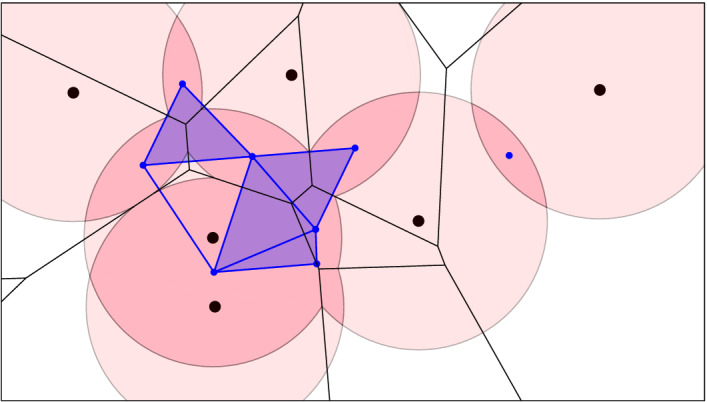
Six points in the plane and a pink ball of radius *r* centered at each. The black order-2 Voronoi tessellation decomposes the twofold cover into convex pieces. The corresponding subcomplex of the dual order-2 Delaunay mosaic is superimposed in blue

Before we discuss this algorithm in Sect. 4, we note that the order-*k* Voronoi tessellation decomposes the *k*-fold cover into convex sets. To see this, let ${|{Q}|} = k$ and define ${{\,\mathrm{dom}\,}}{(Q,r)} = {{\,\mathrm{dom}\,}}{Q} \cap {\mathrm{Cover}_{k}{({Q},{r})}}$, which is an intersection of convex sets and therefore convex. We write ${\mathrm{Vor}_{k}{({X,r})}}$ for the collection of domains ${{\,\mathrm{dom}\,}}{(Q,r)}$ with ${|{Q}|} = k$, and since ${{\,\mathrm{dom}\,}}{(Q,r)} = {{\,\mathrm{dom}\,}}{Q} \cap {\mathrm{Cover}_{k}{({X},{r})}}$, we refer to this as the *Voronoi decomposition* of ${\mathrm{Cover}_{k}{({X},{r})}}$. Since ${{\,\mathrm{dom}\,}}{(Q,r)} \subseteq {{\,\mathrm{dom}\,}}{Q}$, the dual of this decomposition is a subcomplex of the order-*k* Delaunay mosaic, which we denote ${\mathrm{Del}_{k}^{}{({X,r})}} \subseteq {{\,\mathrm{Del}\,}}_{k}{X}$. See Figs. 4 and 6 for example. Modulo a technicality caused by the mosaic not necessarily being simplicial, the Nerve Theorem [17] implies that the cover and the mosaic have the same homotopy type. We shall state and prove this fact more formally now.

### Lemma 3.1

 (Almost Nerve) Let $X \subseteq {{{\mathbb {R}}}}^d$ be locally finite and in general position. For every integer $k \ge 1$ and real $r \ge 0$, ${\mathrm{Del}_{k}^{}{({X,r})}}$ and ${\mathrm{Cover}_{k}{({X},{r})}}$ have the same homotopy type.

### Proof

Recall that the sets ${{\,\mathrm{dom}\,}}{(Q,r)}$ with ${|{Q}|} = k$ form a convex cover of ${\mathrm{Cover}_{k}{({X},{r})}}$. The *nerve* of this collection consists of all sub-collections with non-empty common intersections. By the classic Nerve Theorem [17], this nerve has the same homotopy type as ${\mathrm{Cover}_{k}{({X},{r})}}$, but this does not complete the proof because ${\mathrm{Del}_{k}^{}{({X,r})}}$ is not always isomorphic to the nerve of the ${{\,\mathrm{dom}\,}}{(Q,r)}$. In particular, if an *i*-dimensional Voronoi polyhedron belongs to $m+1\ge d+1-i$ domains, then it maps to an *m*-simplex in the nerve and to an $(d-i)$-cell in the Delaunay mosaic. To make up for the difference, we establish a continuous surjection from the *m*-simplex to the $(d-i)$-cell that extends to a continuous surjection from the nerve to ${\mathrm{Del}_{k}^{}{({X,r})}}$. To describe this surjection, we note that the vertices of the nerve are the ${{\,\mathrm{dom}\,}}{(Q,r)}$, while the vertices of ${\mathrm{Del}_{k}^{}{({X,r})}}$ are the points $x_Q = \sum _{x \in Q} x$. We map each ${{\,\mathrm{dom}\,}}{(Q,r)}$ to $x_Q$ and extend this map linearly to the simplices in the nerve. The resulting map is continuous by construction. To see that it is surjective, we observe that every cell in ${\mathrm{Del}_{k}^{}{({X,r})}}$ corresponds to a simplex whose vertices map to all vertices of the cell. $\square$

**Fig. 5:**
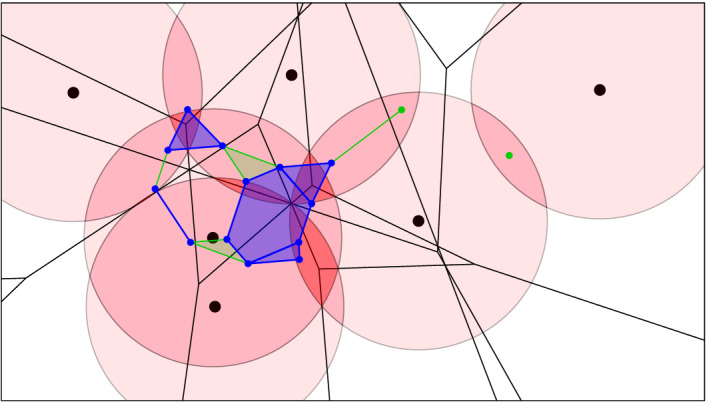
The same point set *X* with balls of radius *r*, and the threefold cover highlighted in darker pink. In black, the degree-3 Voronoi tessellation, which is the superposition of the order-2 and order-3 Voronoi tessellations. It decomposes the threefold cover into convex pieces. In blue, the dual of this decomposition, $D_{2.5}:={\mathrm{Del}_{2.5}^{}{({X,r})}}$. The additional green cells are part of $E_{2.5}\supseteq D_{2.5}$

**Fig. 6:**
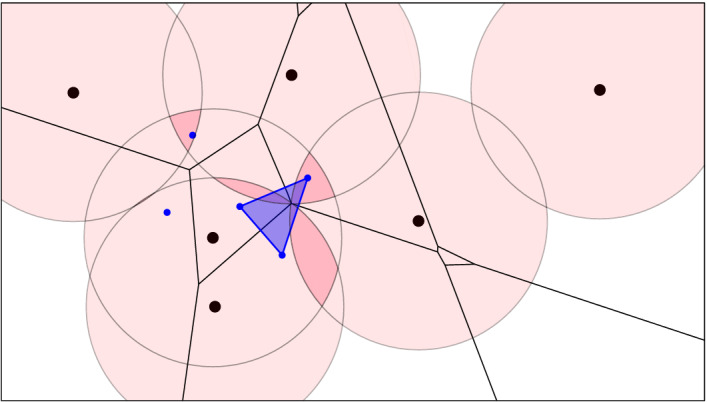
Like Fig. 4, but with threefold cover, order-3 Voronoi tessellation and dual ${\mathrm{Del}_{3}^{}{({X,r})}}$

*The radius function on the rhomboid tiling*   To shed additional light on the subcomplexes of the Delaunay mosaics, we introduce a discrete function on the collection of rhomboids discussed in Sect. 2. Calling it the *radius function*, ${{{\mathscr {R}}}}:{{\,\mathrm{Rho}\,}}{X} \rightarrow {{{\mathbb {R}}}}$, we define it by remembering that each *j*-dimensional rhomboid, ${\rho }\in {{\,\mathrm{Rho}\,}}{X}$, corresponds to a $(d+1-j)$-dimensional cell, ${\rho }^* \in {{\,\mathrm{Arr}\,}}{X}$. Decomposing a point of the cell into its first *d* coordinates and its $(d+1)$-st coordinate, we write $q = (p, z) \in {{{\mathbb {R}}}}^d \times {{{\mathbb {R}}}}$, and we define $r (q) = {\Vert {p}\Vert }^2 - 2z$. With this notation, we define the radius function by mapping ${\rho }$ to the minimum value of any point in its dual cell:

$$\begin{aligned} {{{\mathscr {R}}}}({\rho })=\min _{q \in {\rho }^*} r (q). \end{aligned}$$

By convention, the value of the vertex that corresponds to the ordered three-partition $X = (\emptyset , \emptyset , X)$ is ${{{\mathscr {R}}}}(0) = -\infty$. To obtain a geometric interpretation of this construction, consider the paraboloid defined by the equation $z ={\Vert {p}\Vert }^2/2$ in ${{{\mathbb {R}}}}^{d+1}$ and introduce $\pi _t (p) :{{{\mathbb {R}}}}^d \rightarrow {{{\mathbb {R}}}}$ defined by $\pi _t (p) = ({\Vert {p}\Vert }^2 - t)/2$. The graph of $\pi _t$ is the original paraboloid dropped vertically down by a distance *t*/2. With this notation, ${{{\mathscr {R}}}}({\rho })$ is the minimum *t* such that the graph of $\pi _t$ has a non-empty intersection with ${\rho }^*$.

Clearly, ${{{\mathscr {R}}}}$ is *monotonic*, that is: ${{{\mathscr {R}}}}(\psi ) \le {{{\mathscr {R}}}}({\rho })$ if $\psi$ is a face of ${\rho }$. Indeed, if $\psi$ is a face of ${\rho }$, then ${\rho }^*$ is a face of $\psi ^*$, which implies that the paraboloid touches $\psi ^*$ at the same time or before it touches ${\rho }^*$ when dropped. It follows that the sublevel sets of the radius function are subcomplexes of the rhomboid tiling. For *X* in general position, the radius function satisfies the stronger requirement of a generalized discrete Morse function; see [12, 13]. To explain what this means, let $f :{{\,\mathrm{Rho}\,}}{X} \rightarrow {{{\mathbb {R}}}}$ and for each $r \in {{{\mathbb {R}}}}$ consider the *Hasse diagram*, defined as the graph whose nodes are the rhomboids in $f^{-1} (r)$, with an arc connecting two nodes if one rhomboid is a co-dimension 1 face of the other. The *steps* of *f* are the components of the graphs representing the level sets of *f*. Note that the steps partition ${{\,\mathrm{Rho}\,}}{X}$. We call *f* a *generalized discrete Morse function* if each step is an *interval*, meaning there are rhomboids $\psi \subseteq {\rho }$ such that the step consists of all rhomboids that are faces of ${\rho }$ and contain $\psi$ as a face. It is useful to distinguish between *singular intervals*, when $\psi = {\rho }$, and *non-singular intervals*, when $\psi$ is a proper face of ${\rho }$. Indeed, consider two contiguous sublevel sets that differ by a level set: $f^{-1} [-\infty , r] \setminus f^{-1} [-\infty , r) = f^{-1} (r)$. If this difference is a non-singular interval, then the two sublevel sets have the same homotopy type, while if the difference is a singular interval, then they have different homotopy types. We prove that the radius function is a generalized discrete Morse function with the additional property that every sublevel set is contractible. This is an interesting fact by itself, and it is useful in constructing the radius function on the rhomboid tiling.

### Lemma 3.2

(generalized discrete Morse)  Let $X \subseteq {{{\mathbb {R}}}}^d$ be locally finite and in general position. Then ${{{\mathscr {R}}}}:{{\,\mathrm{Rho}\,}}{X} \rightarrow {{{\mathbb {R}}}}$ is a generalized discrete Morse function. Furthermore, all intervals in the implied partition have a vertex as a lower bound, and there is only one singular interval, which contains the vertex at the origin.

### Proof

The vertex at the origin corresponds to the three-partition $(\emptyset , \emptyset , X)$, has radius ${{{\mathscr {R}}}}(0) = -\infty$, and forms a singular interval. Every other interval is defined by a point $q \in {{{\mathbb {R}}}}^{d+1}$ at which the dropping paraboloid first touches a cell of the arrangement. There is one such point on every plane that is the common intersection of hyperplanes forming the arrangement. By general position, all these points are different. Let *q* belong to an *i*-plane, which is common to $j = d+1-i$ hyperplanes. It belongs to the interior of an *i*-cell, which is common to $2^j$ chambers. Exactly one of these chambers has not already been touched before the *i*-cell. The paraboloid touches this chamber at the same point *q* and, similarly, every cell that is a face of this chamber and contains the *i*-cell as a face. The corresponding rhomboids form an interval of the radius function, with an upper bound of dimension *j* and a lower bound of dimension 0. We have $1 \le j \le d+1$, which implies that the interval is not singular.

To show that ${{{\mathscr {R}}}}$ is a generalized discrete Morse function, we still need to make sure that intervals in the same level set are *separated*, by which we mean that no simplex of one interval is a face of a simplex in the other interval. By the assumption of general position, there is only one level set that contains more than one interval, namely ${{{\mathscr {R}}}}^{-1} (0)$. All its intervals are of the form $[y_x, 0y_x]$, in which *x* is a point in *X*, the origin $0 \in {{{\mathbb {R}}}}^{d+1}$ corresponds to the three-partition $(\emptyset , \emptyset , X)$, and $0 y_x$ is the edge that connects 0 with $y_x$. While these edges all share 0, no two also share the other endpoint. It follows that these intervals are components of the Hasse diagram of the level set, as required. $\square$

*The radius function on a Delaunay mosaic*   Recall that the order-*k* Delaunay mosaic of *X* is the horizontal slice of the rhomboid tiling at depth *k* . In other words, every cell of ${{\,\mathrm{Del}\,}}_{k}{X}$ is the horizontal slice of a rhomboid. More formally, for every ${\sigma }\in {{\,\mathrm{Del}\,}}_{k}{X}$ there is a unique lowest-dimensional rhomboid ${\rho }\in {{\,\mathrm{Rho}\,}}{X}$ such that ${\sigma }= {\rho }\cap {{P}_{k}^{}}$, in which ${{P}_{k}^{}}$ is the horizontal *d*-plane defined by $z = -k$. For vertices we have $\mathrm{dim\,}{{\sigma }} = \mathrm{dim\,}{{\rho }} = 0$, and for all higher-dimensional cells we have $\mathrm{dim\,}{{\sigma }} = \mathrm{dim\,}{{\rho }}-1 \ge 1$. The *radius function* on the order-*k* Delaunay mosaic, ${{{\mathscr {R}}}}_k :{{\,\mathrm{Del}\,}}_{k}{X} \rightarrow {{{\mathbb {R}}}}$, is simply the restriction of ${{{\mathscr {R}}}}$ to the horizontal slice: ${{{\mathscr {R}}}}_k ({\sigma }) = {{{\mathscr {R}}}}({\rho })$. Importantly, this definition is consistent with the subcomplexes ${\mathrm{Del}_{k}^{}{({X,r})}} \subseteq {{\,\mathrm{Del}\,}}_{k}{X}$ used to represent the *k*-fold cover of *X* and *r*, but this needs a proof.

### Lemma 3.3

(Delaunay radius function)  Let $X \subseteq {{{\mathbb {R}}}}^d$ be locally finite and in general position. For every integer $k \ge 1$ and every real *r*, we have ${\mathrm{Del}_{k}^{}{({X,r})}} = {{{\mathscr {R}}}}_k^{-1} [-\infty ,r]$.

### Proof

Recall that $\pi _t :{{{\mathbb {R}}}}^d \rightarrow {{{\mathbb {R}}}}$ is defined by $\pi _t (p) = ({\Vert {p}\Vert }^2 - t)/2$. The graph of $\pi _t$ is a paraboloid that intersects ${{{\mathbb {R}}}}^d$ in the sphere with squared radius *t*. More generally, the paraboloid intersects every *d*-plane tangent to the graph of $\pi _0$ in an ellipsoid whose vertical projection to ${{{\mathbb {R}}}}^d$ is a sphere with squared radius *t*. Dropping the paraboloid vertically thus translates into growing balls simultaneously and uniformly centered at the points in *X*. By definition, ${{{\mathscr {R}}}}({\rho })$ is the value $t_0$ of *t* for which the paraboloid touches the dual cell, ${\rho }^*\in {{\,\mathrm{Arr}\,}}{X}$, for the first time. More formally, the set of points $q \in {\rho }^*$ that lie on or above the graph of $\pi _t$ is empty for all $t < t_0$ and non-empty for all $t \ge t_0$.

Let ${\sigma }^*$ be the vertical projection of ${\rho }^*$ to ${{{\mathbb {R}}}}^d$, and assume it is a polyhedron in some Voronoi tessellation of *X*. It belongs to ${{\,\mathrm{Vor}\,}}_{k}{X}$ iff its dual cell, ${\sigma }$, belongs to ${{\,\mathrm{Del}\,}}_{k}{X}$ or, equivalently, if ${{{\mathscr {R}}}}_k ({\sigma })$ is defined. Assuming the latter, ${\sigma }^* \cap {\mathrm{Cover}_{k}{({X},{r})}}$ is empty for all $r < r_0$ and non-empty for all $r \ge r_0$, in which $r_0^2 = t_0 = {{{\mathscr {R}}}}({\rho }) = {{{\mathscr {R}}}}_k ({\sigma })$. By definition, ${\sigma }$ belongs to ${\mathrm{Del}_{k}^{}{({X,r})}}$ iff this intersection is non-empty, which implies ${\mathrm{Del}_{k}^{}{({X,r})}} = {{{\mathscr {R}}}}_k^{-1} [-\infty , r]$ for all $r \in {{{\mathbb {R}}}}$, as required. $\square$

These results facilitate the computation of the persistence of the *k*-fold covers for varying radii. Lemma 3.1 asserts that we can use ${\mathrm{Del}_{k}^{}{({X,r})}}$ as a proxy for ${\mathrm{Cover}_{k}{({X},{r})}}$. Lemma 3.3 provides the recipe for computing the radii of the cells of ${{\,\mathrm{Del}\,}}_{k}{X}$, and thus the sublevel set filtration of ${{\,\mathrm{Del}\,}}_{k}{X}$, whose persistence module is isomorphic to the persistence of ${\mathrm{Cover}_{k}{({X},{r})}}$ for varying radius *r*. Finally, the persistence diagram is obtained from the filtration via the boundary matrix reduction algorithm [8, Chap. VII].

Assuming $X \subseteq {{{\mathbb {R}}}}^d$ is locally finite and in general position, the radius function of the order-1 Delaunay mosaic is known to be a generalized discrete Morse function [3]. This property does not generalize to higher order. For an example consider the triangle in the middle of Fig. 6, whose step consists of the triangle and its three edges but not of the three vertices. This step is not an interval. Nevertheless, we can still classify the steps of ${{{\mathscr {R}}}}_k$ into critical and non-critical types so that each critical step changes the homotopy type of the sublevel set in a predictable manner, and every non-critical step maintains the homotopy type of the sublevel set. The proof of this claim together with an enumeration of the types of steps can be found in [9].

## Persistence in Depth

In this section, we develop an algorithm that computes the persistence of the nested sequence of multi-covers (5). We follow the usual strategy of substituting a complex for each cover, but there are some complications. Specifically, we represent ${\mathrm{Cover}_{k}{({X},{r})}}$ by ${\mathrm{Del}_{k}^{}{({X,r})}}$ and we introduce additional complexes between contiguous Delaunay mosaics to realize the inclusion between the covers.

*Half-integer slices*   There are generally no convenient maps connecting ${{\,\mathrm{Del}\,}}_{k}{X}$ with ${{\,\mathrm{Del}\,}}_{k-1}{X}$. To finesse this difficulty, we use the horizontal *half-integer slice* of the rhomboid tiling at depth $\ell = k - {1}/{2}$:

$$\begin{aligned} {{\,\mathrm{Del}\,}}_{\ell }{X} = {{P}_{\ell }^{}}\cap {{\,\mathrm{Rho}\,}}{X} \end{aligned}$$

for $k \ge 1$. Similarly to the Delaunay mosaic, the half-integer slice is a polyhedral and not necessarily simplicial complex in ${{{\mathbb {R}}}}^d$; see the hexahedral regions in Fig. 5. Not surprisingly, there is a well-known dual, namely the *degree-k Voronoi tessellation* [10], which we denote ${{\,\mathrm{Vor}\,}}_{\ell }{X}$. It refines the order-*k* Voronoi tessellation by decomposing its domains into maximal regions in which every point has the same *k*-th nearest point in *X*. Similarly, the degree-*k* tessellation refines the order-$(k-1)$ tessellation, and indeed ${{\,\mathrm{Vor}\,}}_{\ell }{X}$ is the superposition of ${{\,\mathrm{Vor}\,}}_{k}{X}$ and ${{\,\mathrm{Vor}\,}}_{k-1}{X}$; see Fig. 5. It can be constructed by projecting the *k*-th level in ${{\,\mathrm{Arr}\,}}{X}$ to ${{{\mathbb {R}}}}^d$. Without going into further details, we observe that this level contains every cell of the arrangement whose corresponding ordered three-partition, $X = {{X}_{in }}\cup {{X}_{on }}\cup {{X}_{out }}$, satisfies ${|{{{X}_{in }}}|} \le k-1$ and ${|{{{X}_{in }}}|} + {|{{{X}_{on }}}|} \ge k$. We refer to the decomposition of ${\mathrm{Cover}_{k}{({X},{r})}}$ by ${{\,\mathrm{Vor}\,}}_{\ell }{X}$ as ${\mathrm{Vor}_{\ell }{({X, r})}}$.

Returning to the mosaics, there are natural piecewise linear maps from ${{\,\mathrm{Del}\,}}_{\ell }{X}$ to ${{\,\mathrm{Del}\,}}_{k}{X}$ and to ${{\,\mathrm{Del}\,}}_{k-1}{X}$. Specifically, we get ${{\,\mathrm{Del}\,}}_{\ell }{X} \rightarrow {{\,\mathrm{Del}\,}}_{k}{X}$ by mapping the vertices dual to the regions decomposing ${{\,\mathrm{dom}\,}}{Q} \in {{\,\mathrm{Vor}\,}}_{k}{X}$ to the vertex dual to ${{\,\mathrm{dom}\,}}{Q}$. Symmetrically, we get ${{\,\mathrm{Del}\,}}_{\ell }{X} \rightarrow {{\,\mathrm{Del}\,}}_{k-1}{X}$. However, because such maps lead to complications in the persistence algorithm, we instead use the horizontal slabs of the rhomboid tiling to connect the mosaics via inclusions. To formally define them, write ${{P}_{\ell }^{k}}$ for the points in ${{{\mathbb {R}}}}^{d+1}$ that lie on or between ${{P}_{\ell }^{}}$ and ${{P}_{k}^{}}$. We define *slab mosaics* as intersections of such slabs with the rhomboid tiling. Analogously to $\hbox {Del}_{k}({X,r})$, we also define radius-dependent subcomplexes of these slab mosaics, as well as of half-integer mosaics:

$$\begin{aligned} {\mathrm{Del}_{\ell }^{k}{({X,r})}}&= \{ {\rho }\cap {{P}_{\ell }^{k}} \, | \,{\rho }\in {{\,\mathrm{Rho}\,}}{X}, \,{{{\mathscr {R}}}}({\rho }) \le r \}, \\ {\mathrm{Del}_{\ell }^{}{({X,r})}}&= \{ {\rho }\cap {{P}_{\ell }^{}} \, | \,{\rho }\in {{\,\mathrm{Rho}\,}}{X}, \,{{{\mathscr {R}}}}({\rho }) \le r \}, \\ {\mathrm{Del}_{k-1}^{\ell }{({X,r})}}&= \{ {\rho }\cap {{P}_{k-1}^{\ell }} \,| \,{\rho }\in {{\,\mathrm{Rho}\,}}{X}, \,{{{\mathscr {R}}}}({\rho }) \le r \}. \end{aligned}$$

**Fig. 7:**
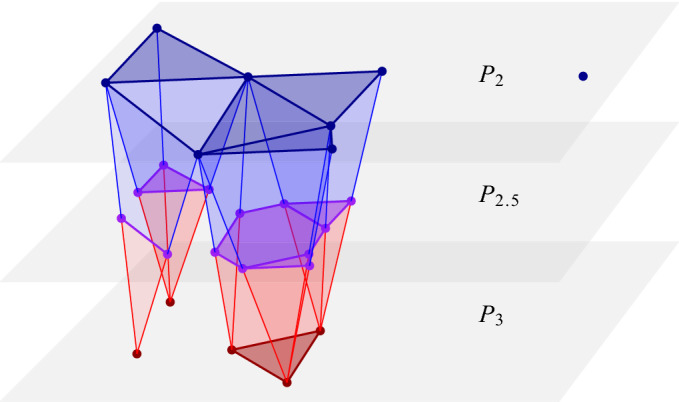
A sublevel set of the 3-dimensional rhomboid tiling of the points in Fig. 4. From *top* to *bottom*: $D_2$ in dark blue, $D_{2.5}$ in purple, and $D_3$ in dark red, with slabs connecting adjacent slices

To simplify the notation, we fix *r* and write $D_k = {\mathrm{Del}_{k}^{}{({X,r})}}$, $C_k = {\mathrm{Cover}_{k}{({X},{r})}}$, etc. The half-integer Delaunay mosaic includes in both slab mosaics, $D_k$ includes in the first and $D_{k-1}$ the second one; see Fig. 7.

We note an important difference between the two slabs: ${\mathrm{Vor}_{\ell }{({X,r})}}$ and ${\mathrm{Vor}_{k}{({X,r})}}$ are different convex subdivisions of the same space, $C_k$, which implies that $D_\ell$ and $D_k$ have the same homotopy type. Indeed, this is also the homotopy type of $D_\ell ^k$, and there are natural deformation retractions to $D_\ell$ and $D_k$. In contrast, $D_{k-1}$ and $D_\ell$ have generally different homotopy types, and there is a deformation retraction from $D_{k-1}^\ell$ to $D_{k-1}$ but not necessarily to $D_\ell$; see again Fig. 7. To remedy this deficiency, we introduce mosaics that contain $D_\ell$ and $D_{k-1}^\ell$ as subcomplexes. To construct them, we recall that ${{\,\mathrm{Vor}\,}}_{\ell }{X}$ is a convex refinement of ${{\,\mathrm{Vor}\,}}_{k-1}{X}$, which implies that the polyhedra of ${{\,\mathrm{Vor}\,}}_{\ell }{X}$ intersect $C_{k-1}$ in convex sets. We let $E_\ell$ be the dual of this convex decomposition of the $(k-1)$-fold cover. Since $C_k \subseteq C_{k-1}$, we indeed have $D_\ell \subseteq E_\ell$; see Fig. 5. Furthermore, we let $E_{k-1}^\ell$ be the maximal subcomplex of ${{P}_{k-1}^{\ell }}\cap {{\,\mathrm{Rho}\,}}{X}$ whose boundary complexes at depths $k-1$ and $\ell$ are $D_{k-1}$ and $E_\ell$. Clearly, $D_{k-1}^\ell$ is a subcomplex of $E_{k-1}^\ell$, and because $D_{k-1}$ and $E_\ell$ are deformation retracts of $E_{k-1}^\ell$, these three mosaics have the same homotopy type. We will use these relations shortly in the computation of the persistence diagram of the filtration of multi-covers (5).

*Connecting the spaces*   To prepare the construction of the persistence and zigzag modules, we connect the multi-covers and the corresponding Delaunay mosaics with maps. Fixing $r \ge 0$ and setting $\ell = k-{1}/{2}$, as before, we consider the following diagram in which identities and homotopy equivalences are marked as such: 
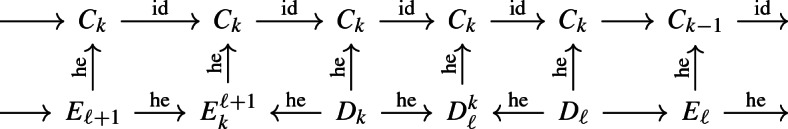


The top row stretches out the filtration by writing each multi-cover five times and connecting the copies with the identity. The remaining maps in this row are inclusions. The bottom row contains the slice mosaics at integer and half-integer depths, and connects them with inclusions, using slab mosaics as intermediaries. As argued above, the first five mosaics all have the same homotopy type, and the inclusion maps between them are homotopy equivalences.

**Fig. 8:**
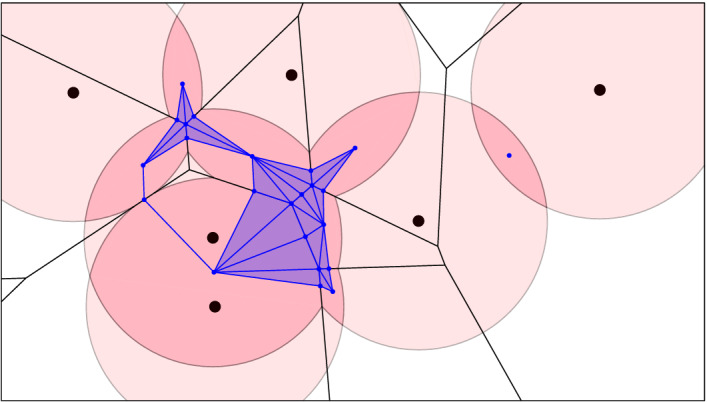
Subdivision of $\hbox {Del}_{2}({X,r})$ with its embedding in ${\mathrm{Cover}_{2}{({X},{r})}}$

To get the vertical map from $D_k$ to $C_k$, we first construct the barycentric subdivision, ${{\,\mathrm{Sd}\,}}{D_k}$, which is a simplicial complex. Each vertex $u\in {{\,\mathrm{Sd}\,}}{D_k}$ represents a *j*-cell in $D_k$, which is dual to a $(d-j)$-dimensional Voronoi polyhedron, and we map *u* to the center of mass of the intersection of this polyhedron with the *k*-fold cover. By construction, this intersection is non-empty and convex, so it contains the center of mass in its interior. After mapping all vertices, we map the other simplices of ${{\,\mathrm{Sd}\,}}{D_k}$ by piecewise linear interpolation; see Fig. 8. The resulting map is injective, and since $D_k$ and $C_k$ have the same homotopy type, the map is a homotopy equivalence. Recall that $D_\ell$ is dual to ${\mathrm{Vor}_{\ell }{({X,r})}}$, which is another convex decomposition of the *k*-fold cover. We therefore get the vertical map from $D_\ell$ to $C_k$ the same way, first constructing ${{\,\mathrm{Sd}\,}}{D_\ell }$ and second mapping the vertices to centers of mass. This is again a homotopy equivalence. Similarly, $E_\ell$ is dual to the convex decomposition of $C_{k-1}$ with ${{\,\mathrm{Vor}\,}}_{\ell }{X}$. As before, we get the vertical map by sending the vertices of $E_\ell$ to centers of mass, but we distinguish between two cases. If a polyhedron of ${{\,\mathrm{Vor}\,}}_{\ell }{X}$ has a non-empty intersection with $C_k$, we send the corresponding vertex of ${{\,\mathrm{Sd}\,}}{E_\ell }$ to the center of mass of this intersection. If, however, the intersection with $C_k$ is empty but the intersection with $C_{k-1}$ is non-empty, then we send the vertex to the center of mass of the latter. This ensures that the geometric embedding of ${{\,\mathrm{Sd}\,}}{D_\ell }$ is contained in the geometric embedding of ${{\,\mathrm{Sd}\,}}{E_\ell }$.

To finally map the slab mosaics, we first deformation retract them to slice mosaics and then map them reusing the barycentric subdivisions. Here we make arbitrary choices, mapping $E_k^{\ell +1}$ to $E_{\ell +1}$ to $C_k$ and mapping $D_\ell ^k$ to $D_k$ to $C_k$. Note that all vertical maps are homotopy equivalences, as marked in the above diagram. We note that not all squares in the diagram of spaces commute. As we will see shortly, however, all squares commute after applying the homology functor, which suffices for our purposes.

*Modules*   Applying the homology functor for a fixed coefficient field, we map all multi-covers and mosaics to vector spaces and all maps to homomorphisms (linear maps) between them. The top row of vector spaces with homomorphisms from left to right is referred to as a *persistence module*, and we denote it $\textsf {MC}({r})$. The bottom row of vector spaces are connected by homomorphisms going from left to right or from right to left. This kind of structure is referred to as a *zigzag module*, and we denote it $\textsf {ZZ}({r})$. The advantage of the zigzag over the persistence module is that its maps are induced by inclusions between complexes, which lend themselves to computations. Our goal, however, is to compute the persistence diagram of $\textsf {MC}({r})$, and we do this by using $\textsf {ZZ}({r})$ as a proxy. The following result is therefore essential.

### Lemma 4.1

(isomorphism of modules)  Let $X \subseteq {{{\mathbb {R}}}}^d$ be locally finite and in general position. Then the persistence diagrams of ${\mathsf {MC}}({r})$ and of ${\mathsf {ZZ}}({r})$ are the same for every $r \ge 0$.

### Proof

Write $\textsf {C}_k,\textsf {D}_k,\textsf {E}_k$ for the vector spaces obtained by applying the homology functor to $C_k,D_k, E_k$, etc. The goal is to show that the diagram of multi-covers and mosaics maps to a diagram of vector spaces in which all squares commute and most maps are isomorphisms: 
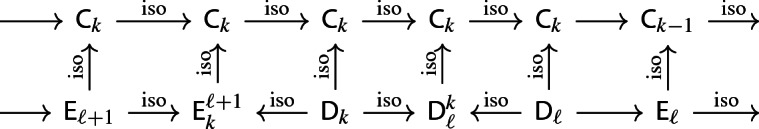


To prove commutativity, we consider the five squares shown in the above diagram. The first square commutes already before applying the homology functor, and so does the third square. Similarly, the fifth square commutes because the image of ${{\,\mathrm{Sd}\,}}{D_\ell }$ in $C_k$ includes in the image of ${{\,\mathrm{Sd}\,}}{E_\ell }$ in $C_{k-1}$.

The second and fourth squares do not commute before applying the homology functor, but we argue they do after applying the functor. The two cases are similar, so we focus on the fourth square. Recall that ${\mathrm{Vor}_{k}{({X,r})}}$ and ${\mathrm{Vor}_{\ell }{({X,r})}}$ are two convex decompositions of the same space, which is $C_k$, and that ${\mathrm{Vor}_{\ell }{({X,r})}}$ is a refinement of ${\mathrm{Vor}_{k}{({X,r})}}$. $D_k$ and $D_\ell$ are dual to these decompositions, with one or more vertices of $D_\ell$ corresponding to every one vertex of $D_k$. When we map $D_\ell$ to $D_\ell ^k$ to $C_k$, the full subcomplex with these vertices is first contracted to the single vertex by the deformation retraction from $D_\ell ^k$ to $D_k$, and second it is mapped to the center of mass of the corresponding domain in ${\mathrm{Vor}_{k}{({X,r})}}$. In contrast, when we map $D_\ell$ to $C_k$ directly, all these vertices map to different points in $C_k$, but all these points lie in the interior of the same domain in ${\mathrm{Vor}_{k}{({X,r})}}$. Indeed, the full subcomplex with these vertices is dual to a convex decomposition of this domain and therefore contractible. It follows that the fourth square of homomorphisms commutes. Similarly, the second square commutes, and therefore all squares commute.

Isomorphisms are reversible, so we can draw them from left to right in the bottom row of the diagram. The result are two parallel persistence modules whose vector spaces are connected by isomorphisms. The Persistence Equivalence Theorem of persistent homology [8, p. 159] implies that the two modules have the same persistence diagram. $\square$

*Algorithm and running time*   We compute the persistence diagram of the filtration of multi-covers (5) using the zigzag algorithm generically described in [4] and explained in detail for inclusion maps in [5]. Its worst-case running time is cubic in the input size, which is the total number of cells in the mosaics. To count the cells, we assume a finite number of points in ${{{\mathbb {R}}}}^d$, $n = {|{X}|}$. All cells are horizontal slices or horizontal slabs of rhomboids in ${{{\mathbb {R}}}}^{d+1}$. We therefore begin by counting the rhomboids or, equivalently, the cells in the dual hyperplane arrangement. These numbers are maximized when the *n* hyperplanes are in general position, and then they depend only on *n* and *d*. Observe first that for every $0 \le i \le d+1$, there are $$\left( {\begin{array}{c}n\\ d+1-i\end{array}}\right) $$ *i*-planes, each the common intersection of $d+1-i$ hyperplanes. There is one chamber for each plane, which implies that the number of chambers in the arrangement is

6 $$\begin{aligned} {{\Gamma }_{d+1}^{d+1}{({n})}}&= \left( {\begin{array}{c}n\\ d+1\end{array}}\right) + \left( {\begin{array}{c}n\\ d\end{array}}\right) + \ldots + \left( {\begin{array}{c}n\\ 0\end{array}}\right) \le \frac{(n+1)^{d+1}}{(d+1)!} . \end{aligned}$$

Indeed, the paraboloid used in the proof of Lemma 3.2 sweeps out the arrangement and encounters a new chamber whenever it first intersects one of the *i*-planes, for $0\le i\le d+1$. The inequality on the right-hand side in (6) is easy to prove, by induction or otherwise. To count the *i*-cells in the arrangement, we observe that each *i*-plane carries an arrangement of $n - (d+1-i)$ $(i-1)$-planes. We get the number of (*i*-dimensional) chambers in this arrangement from (6), and multiplying with the number of *i*-planes, we get the number of *i*-cells:

7 $$\begin{aligned} \begin{aligned} {{\Gamma }_{i}^{d+1}{({n})}}&= \left( {\begin{array}{c}n\\ d+1-i\end{array}}\right) {{\Gamma }_{i}^{i}{({n-d-1+i})}} \\&\le \frac{n^{d+1-i}}{(d+1-i)!}\cdot \frac{(n+1)^i}{i!}\le \frac{(n+1)^{d+1}}{(d+1-i)! \, i!}. \end{aligned} \end{aligned}$$

Writing $j = d-i$, we get a $(j+1)$-rhomboid in ${{\,\mathrm{Rho}\,}}{X}$ for every *i*-cell in the arrangement. In other words, (7) counts the $(j+1)$-rhomboids in the rhomboid tiling. In particular, we have ${{\Gamma }_{d+1}^{d+1}{({n})}}$ vertices in the tiling. For $0 \le j \le d$, the interior of every $(j+1)$-rhomboid has a non-empty intersection with $2j+1$ hyperplanes ${{P}_{\ell }^{}}$, in which $2 \ell$ is an integer. The $(j+1)$-rhomboid thus contributes $2j+1$ *j*-cells to the Delaunay mosaics and $2j+2$ $(j+1)$-prisms to the slab mosaics. Taking the sum over all dimensions, we get the total number of cells in the mosaics used in the construction of the zigzag module:

$$\begin{aligned} \#\,\text {cells}&={{\Gamma }_{d+1}^{d+1}{({n})}}+\sum _{j=0}^d (4j+3){{\Gamma }_{d-j}^{d+1}{({n})}} \\&\le \frac{(n+1)^{d+1}}{(d+1)!}+\sum _{i=0}^d 4(d+1-i)\frac{(n+1)^{d+1}}{(d+1-i)! \,i!} \\&\le \frac{(n+1)^{d+1}}{(d+1)!}+4(n+1)^{d+1}\sum _{i=0}^d\frac{1}{(d-i)!\,i!}\le 9(n+1)^{d+1} . \end{aligned}$$

Taking the third power, we get an upper bound for the worst-case running time of the algorithm and thus the main result of this section.

### Theorem 4.2

(multi-cover persistence)  Let *X* be a set of *n* points in general position in ${{{\mathbb {R}}}}^d$. For every radius $r \ge 0$, the persistence diagram of the filtration of multi-covers with radius *r* can be computed in worst-case time $O(n^{3d+3})$.

## Discussion

The main contribution of this paper is the introduction of the $(d+1)$-dimensional rhomboid tiling of a locally finite set of points in ${{{\mathbb {R}}}}^d$. It is the underlying framework that facilitates the study of multi-covers with Euclidean balls and the computation of the persistence as we increase the radius or we decrease the depth of the coverage. The latter requires novel adaptations of the standard approach to persistence, which for *n* points in ${{{\mathbb {R}}}}^d$ lead to an algorithm with worst-case running time $O(n^{3d+3})$. This compares favorably to naive solutions and the approach using Čech complexes [21], but it is not practical unless *n* and *d* are small. While the time-complexity is too high for the density analysis of large data sets, we see potential applications in the study of regular or semi-regular configurations that arise in the design and investigation of materials. With some modifications, our results extend to balls with different radii (points with weights), see [9], but the implied loss of intuitive appeal prevents us from discussing this generalization. In particular, Theorem 2.1 extends, and Theorem 4.2 holds without change, in this more general setting. There are a number of challenging questions raised by the work reported in this paper.

- Instead of computing the persistence in scale and depth separately, it might be interesting to combine both to a concrete setting for 2-parameter persistence [18].
- A set of *n* points in ${{{\mathbb {R}}}}^d$ has some constant times $n^{d+1}$ ordered three-partitions defined by spheres. We cannot improve the worst-case time of our persistence in depth algorithm unless we avoid the enumeration of these partitions. Can this be done?

As proved in [1], for every locally finite $X \subseteq {{{\mathbb {R}}}}^d$, there is a locally finite $Y \subseteq {{{\mathbb {R}}}}^d$ with real weights such that the (order-1) weighted Voronoi tessellation of *Y* is the order-*k* Voronoi tessellation of *X*. However, growing balls uniformly with centers in *X* and growing them according to the weights with centers in *Y* gives different filtrations of the dual Delaunay mosaic. It would be interesting to quantify this difference by bounding the distance between the two persistence diagrams.
